# Effect of intravenous immunoglobulin on the function of Treg cells derived from immunosuppressed mice with *Pseudomonas aeruginosa* pneumonia

**DOI:** 10.1371/journal.pone.0176843

**Published:** 2017-05-08

**Authors:** Junlu Li, Tingsang Chen, Congcong Yuan, Guoqiang Zhao, Min xu, Xiaoyan Li, Jie Cao, Lihua Xing

**Affiliations:** 1 Department of Respiratory Intensive Care Unit (RICU), The First Affiliated Hospital of Zhengzhou University, Zhengzhou, Henan, China; 2 School of basic medical sciences, Zhengzhou University, Zhengzhou, Henan, China; 3 Department of Laboratory, The First Affiliated Hospital of Zhengzhou University, Zhengzhou, Henan, China; Albany Medical College, UNITED STATES

## Abstract

**Aim:**

The present study aimed to investigate the effect of intravenous immunoglobulin (IVIG) on regulatory T (Treg) cells derived from immunosuppressed mice with *Pseudomonas aeruginosa* (PA) pneumonia.

**Methods:**

A total of 108 BALB/c mice were randomly divided into the following groups: control group (Control), immunosuppressed group (IS), PA pneumonia group (PA), PA pneumonia in immunosuppressed group (IS + PA), PA pneumonia with IVIG treatment in immunocompetent group (PA + IVIG) and PA pneumonia with IVIG treatment in immunosuppressed group (IS + PA + IVIG). Each group comprised 18 mice. The combined PA pneumonia in immunosuppressed model and the treatment models were established. The mice in each group were sacrificed at 4, 8, and 24 h time points. The general condition and pathological changes in the lung tissues of the mice were monitored. Reverse transcription-polymerase chain reaction was used to detect the forkhead box P3 (*FOXP3)* mRNA relative expression level in the lung tissues. The enzyme-linked immunosorbent assay was used to detect the serum concentration of active transforming growth factor beta (TGF-β).

**Results:**

No inflammatory response were exhibited in the lung tissues of the mice in Control group and IS group, while varying degrees of acute lung injury were revealed in the mice in PA group, IS + PA group, PA + IVIG group and IS + PA + IVIG group. Lung tissue injury was most apparent at the 8 h time point, and it indicated the greatest effect in IS + PA group. Whereas tissue damages were alleviated in PA + IVIG group and IS + PA + IVIG group compared with IS + PA group. In addition, tissue damage lessened in PA + IVIG group compared with PA group and IS + PA + IVIG group. *FOXP3* mRNA expression levels in the lung tissues and the serum concentration of TGF-β were lower in IS group, PA group, IS + PA group and IS + PA + IVIG group at the 4, 8 and 24 h time points, respectively compared with Control group. *FOXP3* mRNA expression levels decreased in PA + IVIG group at the 4h time point and TGF-β serum concentrations decreased at the 4 and 8h time points compared with Control group, and subsequently increased.

**Conclusions:**

In the immunosuppred model with PA pneumonia, the immune system was greatly compromised. IVIG partially restored the immunosuppressed functions of Treg cells, suppressed the overactivated immune system and ameliorated the development of the disease.

## Introduction

The number of multidrug-resistant pathogens, such as *Pseudomonas aeruginosa* (PA) that can induce acute and chronic lung infection in immunosuppressed and/or immunodeficient patients is increasing annually worldwide [1,2,3]. The patients present with poor prognosis, although they frequently receive treatment with broad-spectrum antibiotics [4]. The underlying mechanisms are not completely understood. A majority of studies demonstrated that the function of the immune system was impaired in patients with autoimmune diseases. During disease recurrence and/or exacerbation stage, the function of regulatory T (Treg) cells is impaired and the dysfunction of the immune system occurs [5]. Treg cells are subsets of CD4+ T cells with specific immunosuppressive function that play an important role in the development of cancer, autoimmune and infectious diseases [6]. Treg cells suppress immune cell activation, inhibit inflammatory cytokine secretion and regulate immune responses via the expression of a specific transcription factor namely, forkhead box P3 (*FOXP3*). During this process Treg cells secrete various anti-inflammatory cytokines, such as transforming growth factor β (TGF-β) [7,8,9]. The defects of the immunosuppression process lead to immune response overactivation and rapid development of inflammatory diseases. The immunomodulatory capacity and anti-inflammatory effects are considered key properties of intravenous immunoglobulin (IVIG) that are encountered in primary immune deficiencies, immune thrombocytopenic purpura (ITP) and Kawasaki disease. The use of IVIG as a therapeutic regimen has been applied to a variety of autoimmune diseases, including systemic lupus erythematosus (SLE), rheumatoid arthritis (RA), Still’s disease and severe infections [10,11,12]. A recent study demonstrated that a combination of IVIG and antibiotics improved the prognosis of severely infected patients [13,14]. IVIG functions rely on two effective structural groups of the immunoglobulin molecule, namely, the F(ab’)_2_ fragment and the Fc fragment. The F(ab’)_2_ fragment mainly mediates receptor inhibition and cytokine production against pathogens and other noxious antigens, while the Fc fragment mediates modulation and/or inhibition of neonatal Fc receptor (FcRn) saturation and Fc gamma receptor (FcγR) expression. The Fc fragment is also related to the modulation of dendritic cells (DCs), the B cell and Treg cell function and the inhibition/scavenging of complement components [15]. IVIG can promote Treg cell function and the corresponding cytokines secretion, by modifying DCs phenotype to tolerogenic activity and promotion of the STAT5 expression. STAT5 is considered an essential molecule in Treg cell generation [16,17,18]. Based on these findings, the present study investigated the effects of IVIG on Treg cells derived from immunosuppressed mice with PA pneumonia, via the detection of *FOXP3* mRNA expression level in lung tissue and the serum TGF-β concentration.

## Materials and methods

The animal experiments were conducted in compliance with the institutional and federal guidelines regarding the use of animals in research and approved by the Animal Center of Zhengzhou University School of Medicine. In addition, the experimental protocols were reviewed and approved by the Medical Research Ethics Committee of the First Affiliated Hospital of Zhengzhou University.

### Grouping and modeling

**Fig 1:**
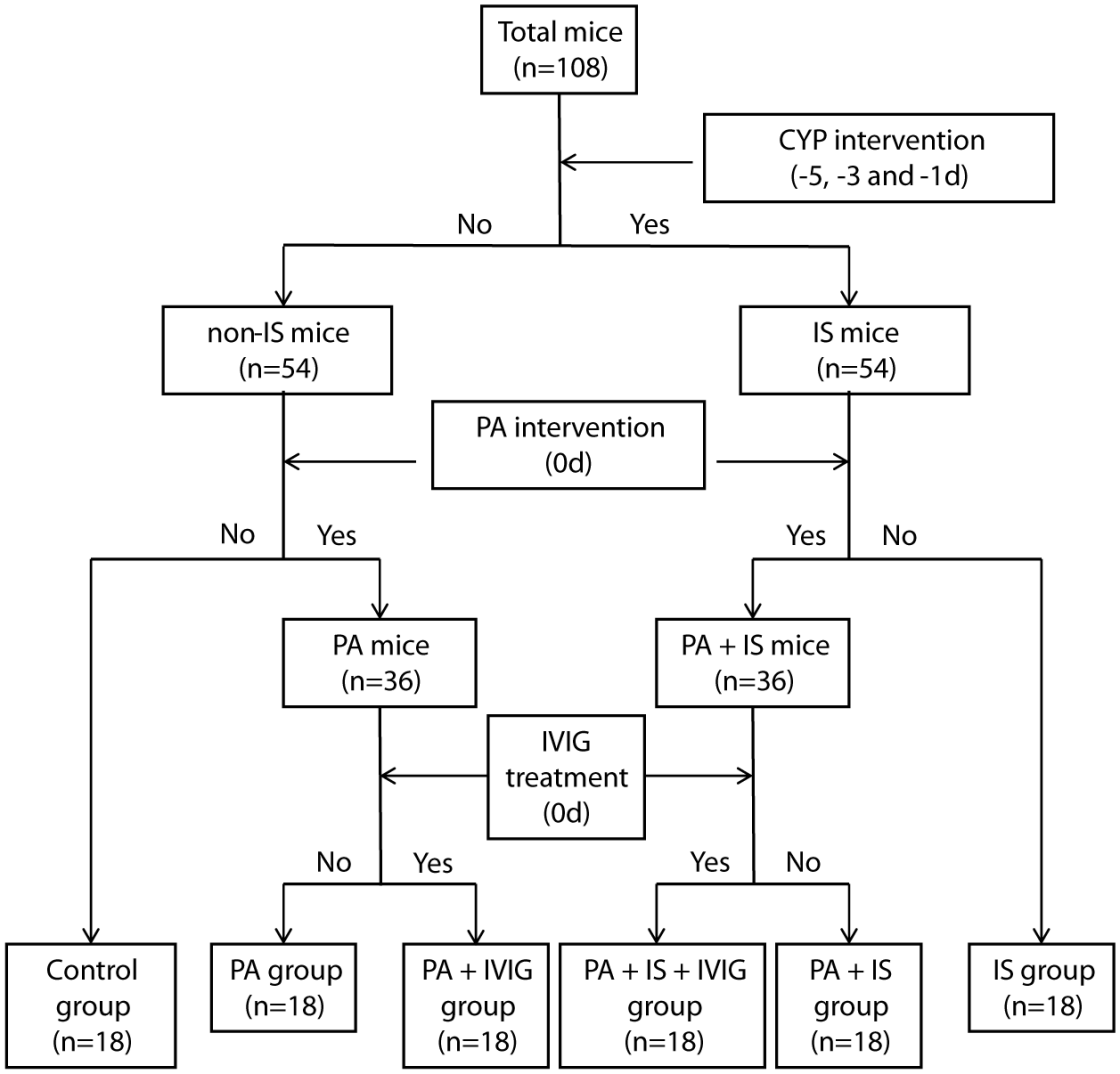
Methods of grouping and modelling.

A total of 108 pathogen-free female BALB/c mice 6 to 8-weeks of age were purchased from the Animal Center of Zhengzhou University, School of Medicine. The animals weighted 25 to 45 g and were randomly divided into six groups, namely, control group (Control), immunosuppresed group (IS), PA pneumonia group (PA), PA pneumonia in immunosuppresed group (IS + PA), PA pneumonia with IVIG treatment in immunocompetent group (PA + IVIG) and PA pneumonia with IVIG treatment in immunosuppresed group (IS + PA + IVIG). All experimental mice were bred and maintained in a sterile animal facility with independent sterile bellows. The immunosuppressed model was established with an intermittent intraperitoneal injection of cyclophosphamide (CYP) (trade name: Endoxan; lot number: H20110407) at a dose of 150 mg per kg, as described in previous studies [19]. The control group received sterile physiological saline on days -5, -3, and -1. PA pneumonia was induced by intranasal instillation with PA suspension (10 μL for each nostril, 10^9^ colony-forming units per mL) on day 0. The control group received sterile phosphate-buffered saline (PBS) suspension on day 0. A combined immunosuppression and PA pneumonia model was established. Following PA intervention on day 0, IVIG—mediated treatment models were immediately established by intraperitoneal injection of mice with 0.4 g per kg of IVIG (lot number: S10970032, purchased from Hualan Biological Engineering Inc). Following successful establishment of the models, six groups of mice were sacrificed at the 4, 8, and 24 h time points with administration of overdose anesthetic by intraperitoneal injection. Methods of grouping and modelling are shown in Fig 1.

### Sample collection

The mice in each group were sacrificed for retro-orbital blood collection (0.8–1 mL). The blood was centrifuged for 15 min at 2,000 rpm. The collected supernatant was stored at –80°C for the estimation of the serum concentration of active TGF-β. Following the collection of the peripheral blood, the mice were disinfected in 75% alcohol. The left lung tissue was collected and stored at –80°C for the detection of the *FOXP3* mRNA relative expression levels by reverse transcription—polymerase chain reaction (RT-PCR). The right lung tissue was fixed in 10% formalin, embedded in paraffin, sliced, and stained with hematoxylin and eosin (H&E) for histological analysis.

### Detection index

#### Pathological score

The right lung of the experimental mice was used for pathological H&E staining and scored by pathologists under a light microscope. Pathological scoring was based on a standard scoring system of acute lung injury [20], including alveolar congestion, pulmonary haemorrhage and/or pulmonary capillary congestion, pulmonary and vessel wall neutrophil accumulation and/or infiltration and alveolar wall thickening and/or hyaline membrane formation. Each side exhibited 5 levels according to the histological assessment: 0 (normal or very minor injuries), 1 (mild injury), 2 (moderate injury), 3 (severe injury) and 4 (very severe damage). The total score ranged between 0 and 20 points.

#### *FOXP3* mRNA expression level

The left lung tissue of the mice was homogenized in RNAase enzyme-free buernisher. Total RNA from lung tissues was extracted using the TRIzol method. It was measured and adjusted to the concentration of 500 ng per μL and the purity of 2.0 according to the A260/A280 ratio. Total cDNA was produced by reverse transcription via a Thermo Fisher reverse transcription kit (purchased from Thermo Fisher Scientific). qRT-PCR was carried out using a Thermo Fisher PCR kit with *FOXP3* and glyceradehyde-3-phosphate-dehydrogenase (GAPDH) primers (synthesized by TaKaRa Biological Engineering Co., Ltd. Shanghai, China). GAPDH was used as an endogenous control. The reaction was carried out in a Thermo Fisher 7500 PCR light cycler. The PCR reaction conditions were as followed: an initial denaturation was conducted at 95°C for 3 min and subsequently 40 cycles of the 3 steps, namely, denaturation at 95°C for 35 s, annealing at 55°C for 35 s and extension at 72°C for 40 s. The final extension was conducted at 72°C for 5 min. The cycle threshold values were obtained for each group and calculated by the 2 ^(- ΔΔ CT)^ method in order to estimate the *FOXP3* mRNA relative expression level. The primer sequences were as followed: GAPDH: forward primer: 5'GCACCGTCAAGGCTGAGAAC3'; reverse primer: 5'TGGTGAAGACGCAGTGGA3'; *FOXP3*: forward primer: 5'CCACTCCAGACAGAAGAAAGC 3'; reverse primer: 5'TCCAAGTCTCGTCTGAAGGC3 '.

#### TGF-β serum concentration

The serum samples that were stored at –80°C were thawed at room temperature and the supernatant was collected following centrifugation at 3,000 rpm for 5 min. The samples were heated in a water bath at 80°C for 8 min and cooled for 5 min. The kit was kept at room temperature for 30 min, according to the manufacturer’s instructions (purchased from Dake Biotechnology Co., Ltd.) in order to estimate the serum concentration of active TGF-β. Each sample was tested in triplicate. The fluorescence of each well was measured using a microplate reader. The serum concentration of active TGF-β was determined by comparison with a standard curve.

### Statistical analysis

Data were analyzed using SPSS17.0 (SPSS, IL, USA). The values were expressed as mean ± SD. The quantification data were assessed using the least significant difference (LSD) method of one-way analysis of variance and Bonferroni’s test was carried out for the comparison between different groups. A *P* value of less than 0.05 (*P*<0.05) was considered statistically significant.

## Results

### General condition and manifestation

The general condition of the mice did not change significantly following the induction of the models (including mental status, weight, activities, and the amount of food and drinking water) in Control group and IS group. And the lung tissue was pink and shiny with no bleeding or pus observed by visual investigation in the mice in Control group and IS group. But varying degrees of listlessness, reduced activities, reduced food and water consumption, weight loss, and yellow color of the coat were indicated in the mice in PA group, IS + PA group, PA + IVIG group and IS + PA + IVIG group. Their lung tissue was dark and reluster. Varying degrees of bleeding and pus were visible on the surface of lung in PA group, IS + PA group, PA + IVIG group and IS + PA + IVIG group. Whereas greater severity in terms of tissue damage were indicated in mice in IS + PA group.

### Histopathological analysis of lung tissue

**Fig 2:**
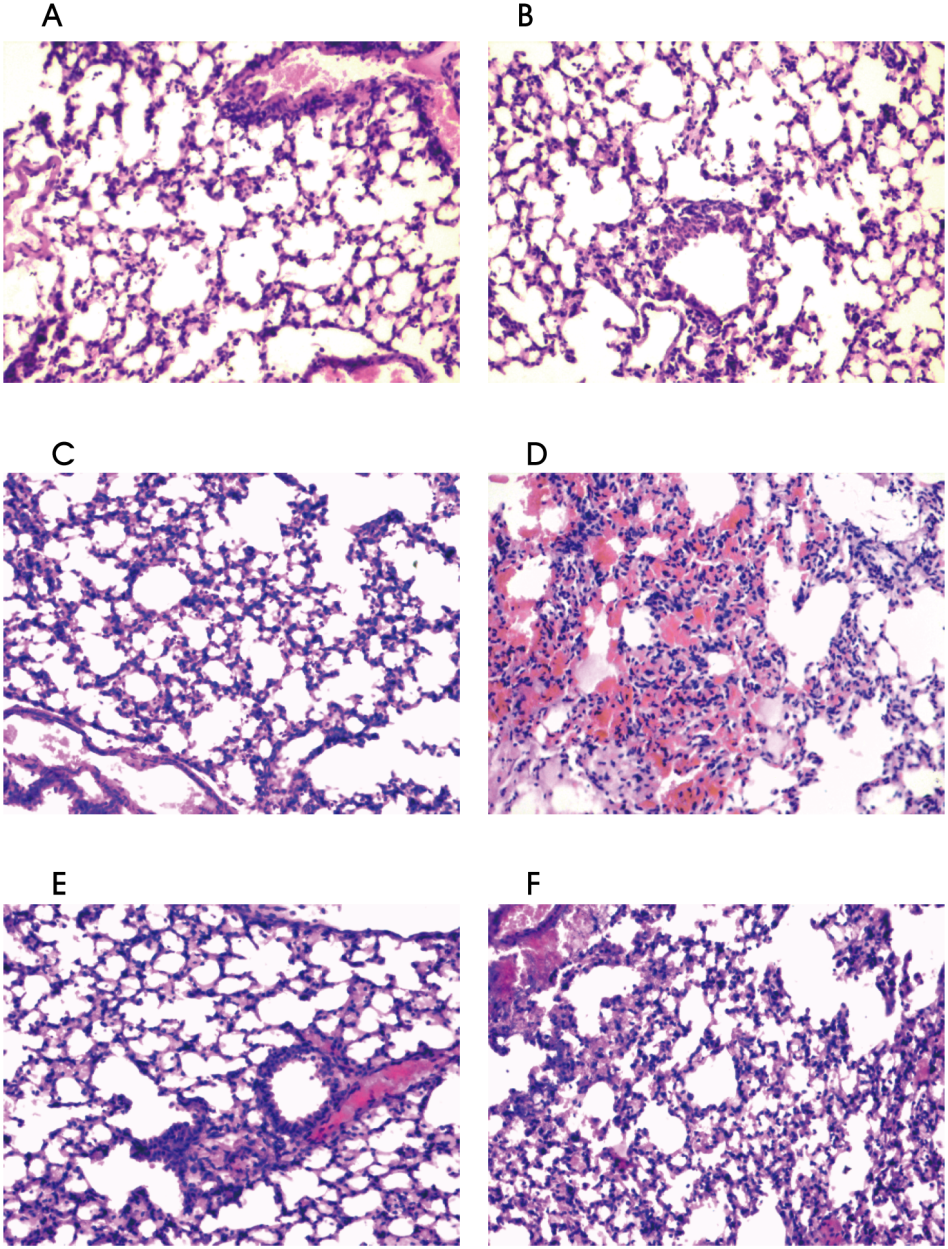
Lung histopathological manifestations of the lung tissues at 8 h in each group. Six groups were studied: Control group (A), IS group (B), PA group (C), IS + PA group (D), PA + IVIG group (E) and IS + PA + IVIG group (F). Immunosuppression was induced on day -5, -3 and -1 by intraperitoneal injection of CYP, PA pneumonia was induced on day 0 and IVIG mediated treatment models were immediately established following PA intervention on day 0. Following establishment of the models, mice were sacrificed at the 4, 8, and 24 h time points. The right lung tissue was fixed in 10% formalin, embedded in paraffin, sliced, and stained with H&E for histological analysis. Histopathological manifestations of lung tissue in six groups at 8h were shown with magnified 100 times.

**Fig 3:**
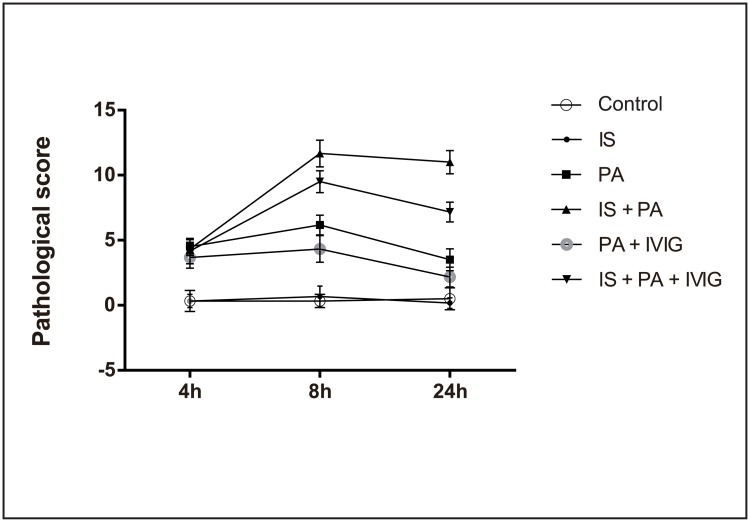
Pathological score of lung tissue in each group at 4, 8 and 24h. Six groups were studied: Control group, IS group, PA group, IS + PA group, PA + IVIG group and IS + PA + IVIG group. Immunosuppression was induced on day -5, -3 and -1 by intraperitoneal injection of CYP, PA pneumonia was induced on day 0 and IVIG mediated treatment models were immediately established following PA intervention on day 0. The right lung of the experimental mice was used for pathological analysis and scored based on a standard scoring system of acute lung injury. Pathological scores of lung tissue in six groups at 4, 8 and 24h were shown.

Light microscopic analysis revealed that normal lung tissue structures without obvious lung injury were exhibited in the mice in Control group and IS group at the three time points (4, 8 and 24 h). However, varying degrees of acute lung injury were exhibited in the mice in PA group, IS + PA group, PA + IVIG group and IS + PA + IVIG group at the 4 h time point. The injury exhibited the greatest severity at the 8 h period, and the most severe injuries were displayed in the mice in IS + PA group. Severe pulmonary capillary congestion, massive pulmonary haemorrhage, neutrophil infiltration, alveolar wall thickening and/or breaking and alveolar hyaline membrane formation were indicated in the mice in IS + PA group. After treatment with IVIG, less lung injury was exhibited in the mice in IS + PA + IVIG group compared with IS + PA group. Capillary congestion, pulmonary bleeding, moderate infiltration of neutrophils and alveolar wall thickening and/or breaking were exhibited in the mice in IS + PA + IVIG group. Similarly, lesser lung injury was indicated in the mice in PA + IVIG group compared with PA group. Apparent capillary congestion, a low-to-moderate amount of red blood cell leakage in the lungs, neutrophil infiltration and partial alveolar wall thickening in the absence of hyaline membrane formation were exhibited in the mice in PA group. Mild lung injury including neutrophil infiltration in several local tissues, a small amount of red blood cell leakage and thickened alveolar walls were exhibited in the mice in PA + IVIG group. The acute lung damages were alleviated in PA group, PA + IVIG group and IS + PA + IVIG group at the 24 h time point, while a severe injury in the absence of improvement was indicated in IS + PA group. The comparison of different groups at the same time point revealed that with the exception of PA group, IS + PA group, PA + IVIG group and IS + PA + IVIG group at the 4h time point, as well as Control group and IS group at the three time points (4, 8 and 24 h), the differences among the remaining groups were statistically significant (*P* < 0.05). The comparison of the same group at different time points revealed that with the exception of Control group and IS group at the three time points (4, 8 and 24 h), IS + PA group at the 8 and 24 h time points and PA + IVIG group at 4 and 8h time point, the differences observed in the remaining groups were statistically significant (*P* < 0.05). The lung histopathological manifestations of the lung tissues at 8 h in each group are shown in Fig 2, and the pathological scores in each group are shown in Fig 3.

### *FOXP3* mRNA relative expression levels in lung tissues

**Fig 4:**
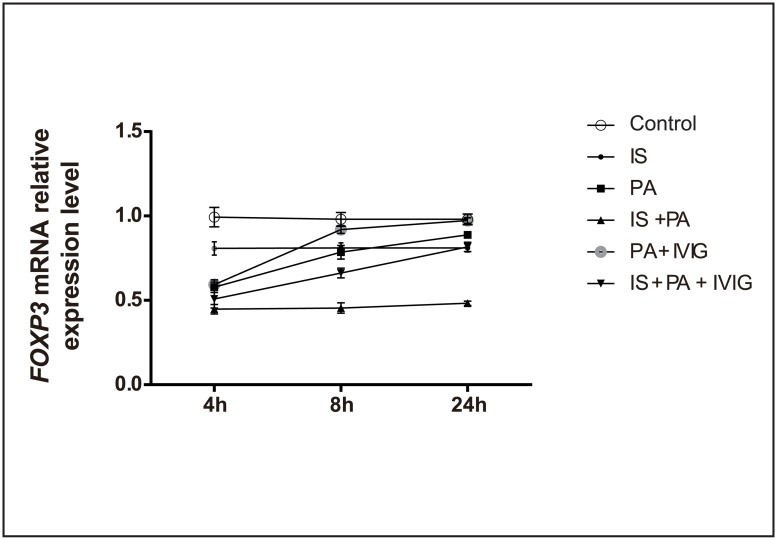
*FOXP3* mRNA relative expression level in lung tissue in each group at 4, 8 and 24h. Six groups were studied: Control group, IS group, PA group, IS + PA group, PA + IVIG group and IS + PA + IVIG group. Immunosuppression was induced on day -5, -3 and -1 by intraperitoneal injection of CYP, PA pneumonia was induced on day 0 and IVIG mediated treatment models were immediately established following PA intervention on day 0. The left lung tissue of the mice was used for testing *FOXP3* mRNA relative expression by qRT-PCR. qRT-PCR was carried out using a Thermo Fisher PCR kit with FOXP3 and GAPDH primer. *FOXP3* mRNA relative expression level was calculated by the 2 ^(- ΔΔ CT)^ method. *FOXP3* mRNA expression levels in lung tissue in six groups at 4, 8 and 24h were shown.

*FOXP3* mRNA relative expression levels did not show significant changes in Control group at the three time points (4, 8 and 24 h). However, *FOXP3* mRNA relative expression levels were down-regulated in IS group, PA group, IS + PA group, PA + IVIG group and IS + PA + IVIG group at the 4 h time point. This effect was greater in IS + PA + IVIG group and lesser in IS group. *FOXP3* mRNA levels tended to increase in PA group, PA + IVIG group and IS + PA + IVIG group, with the exception of IS group and IS + PA group at the 8 and 24 h time points. *FOXP3* mRNA relative expression levels were indicated down-regulated in IS group at the three time points compared with Control group. This demonstrated that CYP may inhibit Treg cellular proliferation and suppressive function. However, *FOXP3* mRNA relative expression levels were lower in IS + PA group compared with IS group at the 4, 8 and 24 h time points, which suggested that the down-regulation of Treg cells in immunosuppressed mice with PA pneumonia was notably associated with the impaired immune system, rather than with the intervention of CYP alone. Following treatment with IVIG, *FOXP3* mRNA relative expression levels were higher in IS + PA + IVIG group compared with IS + PA group at 8 and 24h. Similarly, higher *FOXP3* mRNA relative expression levels were indicated in PA + IVIG group compared with PA group at 8 and 24h. The data suggested that IVIG modulated the immune response by promoting Treg cells function and/or proliferation. The comparison of different groups at the same time point demonstrated that the differences exhibited statistical significance (*P* < 0.05), with the exception of the following: PA group and IS + PA + IVIG group, PA group and PA + IVIG group, IS + PA group and IS + PA + IVIG group at the 4 h time point, PA group and IS group at the 8 h time point, Control group and PA + IVIG group, as well as IS group and IS + PA + IVIG group at the 24 h time point. The comparison in the same group at different time points revealed that with the exception of Control group and IS group at the three time points (4, 8 and 24 h) and IS + PA group at 4 and 8h time points, the differences were statistically significant for the remaining groups (*P* < 0.05). The *FOXP3* mRNA relative expression levels in the lung tissues are shown in Fig 4.

### Serum concentration of active TGF-β

**Fig 5:**
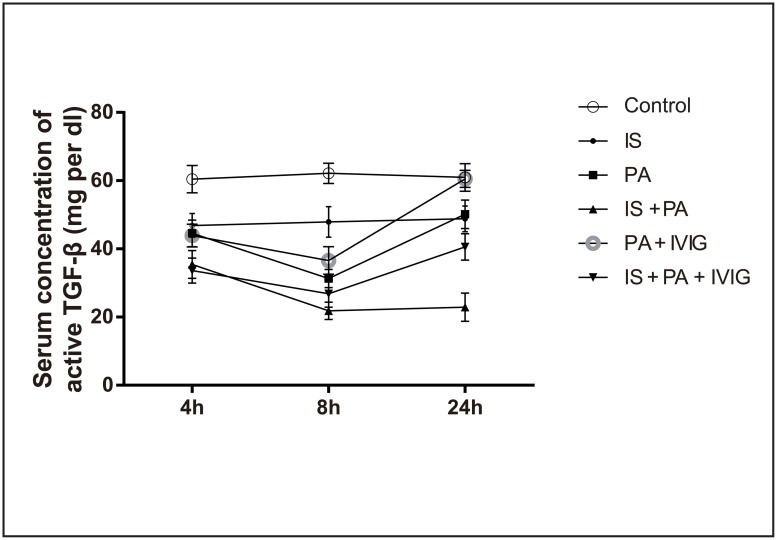
Serum concentrations of active TGF-β in each group at 4, 8 and 24h. Six groups were studied: Control group, IS group, PA group, IS + PA group, PA + IVIG group and IS + PA + IVIG group. Immunosuppression was induced on day -5, -3 and -1 by intraperitoneal injection of CYP, PA pneumonia was induced on day 0 and IVIG mediated treatment models were immediately established following PA intervention on day 0. Serum samples were heated in a water bath at 80°C for 8 min and cooled for 5 min. The serum concentration of active TGF-β was tested by ELISA kit and was determined by comparison with a standard curve. Serum concentrations of active TGF-β in six groups at 4, 8 and 24h were shown.

TGF-β can be secreted in latent or active forms, although the serum concentration of latent TGF-β is considerably low. The serum concentration of active TGF-β was tested following heating of the samples to induce activation of the TGF-β protein. The serum concentration of TGF-β in the peripheral blood exhibited no apparent change in Control group at the three time points. The concentrations of TGF-β were reduced in IS group, PA group, IS + PA group, PA + IVIG group and IS + PA + IVIG group compared with that noted in Control group at the 4 and 8 h time points. An increase was demonstrated at the 24 h time point in PA group, PA + IVIG group and IS + PA + IVIG group. The increased concentration of TGF-β was considerably high in PA + IVIG group, while it lessened in PA group and IS + PA group. The down-regulated TGF-β concentration in IS group did not change at the three time points, indicating the persistent suppression of Treg cells by CYP. However, the concentrations of TGF-β were considerably lower in IS + PA group compared with that noted in IS group at the three time points. This finding indicated that the suppression of Treg cell function in immunosuppressed host with PA pneumonia was notably associated with an impairment of the immune system, rather than a pharmacological effect caused by intervention of the animals with CYP alone. In addition, the concentration of TGF-β was higher in IS + PA + IVIG group compared with IS + PA group at the 24h time point, whereas higher concentration of TGF-β were exhibited in PA + IVIG group compared with PA group at the 24h time point, following treatment with IVIG. These findings indicated that IVIG may improve Treg cell function. The comparison of the different groups at the same time point demonstrated that with the exception of the 4 h time point in IS + PA group and IS + PA + IVIG group, the 4 h time point in PA group, IS group and PA + IVIG group, the 8h time point in PA group and IS + PA + IVIG group, the 8h time point in PA group and PA + IVIG group, the 8 h time point in IS + PA group and IS + PA + IVIG group, the 24 h time point in Control group and PA + IVIG group and the 24 h time point in PA group and IS group, differences among the remaining groups were statistically significant (*P* < 0.05). The comparisons of the same groups at different time points indicated that with the exception of Control group and IS group at the three time points and IS + PA group at the 8 and 24 h time points, the differences noted were statistically significant for the remaining groups (*P* < 0.05). The serum concentrations of active TGF-β are shown in Fig 5.

## Discussion

Treg cells were identified as subsets of CD4 + T cells [21], which play important roles in the regulation of immune tolerance, immunosuppression, development of inflammation and tumour [7]. Treg cells can secrete a large amount of anti-inflammatory cytokines such as, IL-10, TGF-β and IL-35. TGF-β is one of the major effectors of Treg cells. It is also secreted by B cells, macrophages, and DCs. It promotes Treg cell differentiation and *FOXP3* expression. The interaction between TGF-β and *FOXP3* plays an important role in the maintenance of immune tolerance [22]. *FOXP3* is a transcription factor, specifically expressed in Treg cells. It can induce Treg cell differentiation, promote cell proliferation and stabilize the expression of multiple cell surface molecules in T cells express antigen receptors (TCR) -dependent manner. It can also inhibit the secretion of a variety of pro-inflammatory cytokines [9]. To date, a limited number of studies have focused on Treg cell function in pneumonia, notably in pneumonia under immunosuppressed conditions. Treg cells were shown to alleviate lung injury by the modulation of the immune response, although the underlying mechanism remains unclear [23].

IVIG replacement therapy was applied to congenital primary immunodeficiency disease for the first time in the 1950s [24]. IVIG is now widely used in the treatment of autoimmune diseases, primary or secondary immunodeficiency diseases, and severe infections [10,11,25]. The primary action of immunoglobulins mainly relies on two segments, the F(ab’)_2_ and the Fc portion. The F(ab’)_2_ portion mediates inhibition of the receptors, antibodies and cytokines, while the Fc portion is associated with saturation of FcRn, modulation of FcγR expression, modulation of DCs and B cells and inhibition and/or clearance of the complement components [15,26]. Immunoglobulins further promote production of Treg cells and immune-suppressive function. Immunoglobulins may modulate the number of Treg cells by modification of the DC phenotype to immature or tolerogenic DCs and promotion of STAT5 expression, an essential molecule related to the production of Treg cells [17]. They also increase Treg cells suppressive potency by increasing the percentage of Treg cells in the CD4 population and/or by increasing the percentage of Treg cells expressing IL-10, as well as promoting *FOXP3* expression and the secretion of effector molecules [18]. However, the effects of immunoglobulins to Treg cells in the immunosuppressed host with PA pneumonia remain unclear.

CYP is a cytotoxic alkylating agent that is widely used for the treatment of neoplastic disease and severe autoimmune diseases. CYP is widely used in the establishment of the immunosuppressed animal model [27]. Studies on CYP have demonstrated that the compound inhibits Treg cell production and suppressive function by the following modes of action: ablation of the DCs involved in peripheral tolerance, interference with homeostatic proliferation of Treg cells, increase in the susceptibility of Treg cells to apoptosis, decrease of the suppressive effectors of *FOXP3* experssion and reduction of the expression of CD25, a critical factor for the growth of Treg cells [28,29]. Despite the aforementioned findings, the association of the inhibition of Treg cells in immunosuppressed host with PA pneumonia with the effect caused by CYP remains unkown.

*FOXP3* mRNA expression levels in the lung tissues and the concentration of TGF-β in serum, were decreased slightly in the CYP -induced IS mice compared with Control mice (Figs 4 and 5). Furthermore, in PA mice, histopathological analysis exhibited moderate lung injury (Fig 2B). *FOXP3* mRNA expression levels in lung tissues were reduced at the 4 h time point, while they increased at the 8 and 24 h time points (Fig 4). The concentration of TGF-β in serum decreased at the 4 and 8 h time points and increased at the 24 h time point (Fig 5). The increased tendency of *FOXP3* mRNA expression levels and the concentration of TGF-β in the serum indicated that Treg cells in PA mice may be timely activated by the immune system during homeostasis. However, it was observed that PA pneumonia in immunosuppressed mice developed rapidly and was uncontrolled compared with normal mice. PA pneumonia—induced lung injury was severe in IS + PA mice (Fig 2D). In addition, *FOXP3* mRNA expression levels in lung tissues and the concentration of TGF-β in the serum were significantly reduced compared with IS mice and PA mice (Figs 4 and 5). This finding indicated that in IS + PA mice, the population of Treg cells was greatly inhibited due to the disturbed immune system and not due to CYP intervention or PA infection alone. Following 4 h of IVIG treatment, the expression levels of *FOXP3* mRNA in the lung tissues increased (Fig 4). Following 8 h of IVIG treatment, the peripheral blood concentrations of TGF-β increased (Fig 5), and the lung tissue damage was also alleviated (Fig 2E and 2F). Following 24 h of IVIG treatment, the lung injury was considerably alleviated. The results suggested that in the IS + PA mice, the Treg cell function was apparently impaired and the immune responses were disordered. A previous study demonstrated that Th17 cells were overactivated and secreted excessive pro-inflammatory cytokines that resulted in organ damage in the immunosuppressed and PA pneumonia model. However, the immunomodulatory capacity of Treg cells was impaired that in turn strengthened the immune response and promoted the development of the disease. Following IVIG treatment, the activation of Th17 cells was partially inhibited and the immunosuppressive functions of the Treg cells were amplified. Consequently, this process promoted the restoration of the function of the immune system and the alleviation of the development of the disease.

In conclusion, the data demonstrated that the immune system was compromised and the immunomodulatory capacity of the Treg cells was impaired persistently in the immunosuppressed model with PA pneumonia. The overactivation of the immune response without suppression and the infiltration of the immune cells in the lung tissue caused severe tissue damage and resulted in rapid development of the disease. Nevertheless, following IVIG treatment, the immune system impairment was ameliorated and the immunosuppressed functions of Treg cells were partially restored. This process resulted in considerably less tissue damage and promoted disease recovery. Consequently, the use of IVIG in the regulation of the immune response and the restoration of the immunosuppressed function may be a powerful tool to treat immunocompromised patients with infections.

## Supporting information

S1 Data Data. (XLSX)
